# *De novo* malignancy in organ transplant recipients in Taiwan: a nationwide cohort population study

**DOI:** 10.18632/oncotarget.13124

**Published:** 2016-11-04

**Authors:** Hsin-I Tsai, Chao-Wei Lee, Chang-Fu Kuo, Lai-Chu See, Fu-Chao Liu, Meng-Jiun Chiou, Huang-Ping Yu

**Affiliations:** ^1^ Department of Anesthesiology, Chang Gung Memorial Hospital, Taoyuan, Taiwan; ^2^ College of Medicine, Chang Gung University, Taoyuan, Taiwan; ^3^ Graduate Institute of Clinical Medical Sciences, Chang Gung University, Taoyuan, Taiwan; ^4^ Department of Surgery, Change Gung Memorial Hospital, Taoyuan, Taiwan; ^5^ Division of Rheumatology, Allergy and Immunology, Chang Gung Memorial Hospital, Taoyuan, Taiwan; ^6^ Department of Public Health, College of Medicine, Chang Gung University, Taoyuan, Taiwan; ^7^ Biostatistics Core Laboratory, Molecular Medicine Research Center, Chang Gung University, Taoyuan, Taiwan; ^8^ Office for Big Data Research, Chang Gung Memorial Hospital, Taoyuan, Taiwan

**Keywords:** de novo malignancy, organ transplantation, nationwide study, population-based study, cohort study

## Abstract

Organ transplant recipients appear to have a higher risk of de novo malignancy. The aim of the study was designed to estimate cancer risk in heart, lung, kidney and liver transplant recipients. The cohort study used the Taiwan National Health Insurance Research Database (1996-2011) and followed the outcomes of organ recipients until 2012. De novo cancer and mortality rates after organ transplantation were evaluated using standardized incidence ratios, excess absolute risks of cancer, and standardized mortality ratios in recipients were compared with those in the general population. We identified 40, 231, 2, and 115 patients who developed cancer after heart, kidney, lung, and liver transplantation, which corresponded to a cancer incidence of 878.4, 1101.2, 728.9, and 1361.4 cases per 100,000 person-years, respectively. In heart, kidney, lung, and liver recipients, the overall standardized incidence ratios were 1.65 (1.21-2.24), 3.33 (2.93-3.79), 1.82 (0.45-7.27) and 3.37 (2.81-4.05) and the overall standardized mortality ratios were 5.45 (4.96-5.98), 1.47 (1.34-1.61), 8.92 (7.10-11.20), and 3.83 (3.48-4.20), respectively. These results reveal a three-fold increase in de novo cancer risk in organ transplant patients compared with the general population. This study illustrated the importance of de novo malignancy after organ transplantation.

## INTRODUCTION

Solid organ transplantation is an increasingly common measure to rescue life of patients who experience end organ failure. To prevent graft failure, an immunosuppressant is necessary; however, suppressed immune function may fail to contain malignant transformation. Consequently, transplant recipients tend to have a higher risk of developing de novo cancer [1–3].

Recent literature has demonstrated a two- to five-fold increase in the risk of new-onset cancer in transplant recipients [4–7]. The risks are especially high for oncovirus-related cancers. The most common de novo malignancies are non-Hodgkin lymphoma and Hodgkin lymphoma (both due to Epstein-Barr virus [EBV]), Kaposi sarcoma (human herpesvirus 8 [HHV-8]), and liver cancers (hepatitis B virus [HBV], and hepatitis C virus [HCV]), and ano-genital skin cancers (human papillomavirus, [HPV]) [8–11].

A majority of large population-based studies have been conducted in Western countries [9, 12–17] with predominantly Caucasian populations. Although two recent Japanese studies revealed that de novo malignancy occured at an incidence of 10.1% and post transplant lymphoproliferative disorder was the most common malignancy after lung transplantation, regardless the source of organ was from living donor or cadaver [18, 19], the cancer risk among Asian organ transplant recipients has been limited. However, Taiwan launched the National Health Insurance (NHI) Program in 1995, with nationwide coverage. The National Health Insurance Research Database (NHIRD) has nationwide representation, large size, and accurate data on diagnosis of cancer, organ transplant, cohort nature, and long term follow-up, allowing the study of the risk of cancer and mortality in Taiwanese organ transplant recipients. The current study aimed to estimate the incidence rates of de novo cancer and mortality in patients who received heart, kidney, lung, or liver transplantation between 1996 and 2011, and were followed-up until 2012.

## RESULTS

Between 1996 and 2011, 7099 patients underwent organ transplantation, only 6265 of which underwent their procedure in Taiwan. A total of 834 patients had cancer before organ transplantation. Approximately 60% of organ recipients were male, and the mean age at transplant was 42.5 years. The most commonly transplanted organ was the kidney (52.02%), followed by the liver (31.26%), the heart (15.08%), and the lung (1.63%). Except for lung recipients, men predominated in the other three types of organ transplantation (Table 1). Recipients of kidney and liver transplantation appeared to have a higher socioeconomic status than heart and lung recipients. The mortality was significantly higher in patients receiving organ transplantation, with an standardized mortality ratio (SMR) of 2.73 (95% CI, 2.59-2.87); patients receiving lung transplantation were associated with the highest SMR, of 8.92 (95% CI, 7.10-11.20), followed by SMR of 3.83 (95% CI, 3,48-4.2) in liver transplant recipients and 1.47 (95% CI, 1.34-1.61) in kidney transplant recipients.

**Table 1:** Clinical characteristics of patients receiving organ transplantation comparing to at risk population for cancer

Variables	All	Heart/Lung	Kidney	Liver
No.	6265	1047	3259	1959
Age (years) (mean ± standard deviation)	42.50±15.56	45.74±14.73	41.52±12.50	42.39±19.77
Sex				
Male	3752(59.89)	247(23.59)	1647(50.54)	1305(66.62)
Female	2513(40.11)	800(76.41)	1612(49.46)	654(33.38)
Place of residence, No. (%)				
Urban	2102(33.55)	369(35.24)	1202(36.88)	531(27.11)
Suburban	1808(28.86)	284(27.13)	966(29.64)	558(28.48)
Rural	2349(37.49)	394(37.63)	1086(33.32)	869(44.36)
Unknown	6(0.10)	0	5(0.15)	1(0.05)
Income levels, No. (%)				
Quintile 1	1153(18.40)	234(22.35)	578(17.74)	341(17.41)
Quintile 2	701(11.19)	124(11.84)	389(11.94)	188(9.6)
Quintile 3	1689(26.96)	274(26.17)	875(26.85)	540(27.57)
Quintile 4	1262(20.14)	201(19.20)	637(19.55)	424(21.64)
Quintile 5	1387(22.14)	199(19.01)	752(23.07)	436(22.26)
Unknown	73(1.17)	15(1.43)	28(0.86)	30(1.53)
Occupation, No. (%)				
Dependents of the insured individuals	1552(24.77)	234(22.35)	644(19.76)	674(34.41)
Civil servants, teachers, military personnel and veterans	309(4.93)	46(4.39)	179(5.49)	84(4.29)
Non-manual workers and professionals	1566(25.00)	265(25.31)	929(28.51)	372(18.99)
Manual workers	2189(34.94)	346(33.05)	1182(36.27)	661(33.74)
Other	649(10.36)	156(14.90)	325(9.97)	168(8.58)
Person-years of follow-up (years)	35363	4898	21751	8714
Died (per 1000 person-year)	1415 (40.01)	513 (104.74)	472 (21.70)	430 (49.35)
SMR (95% CI)	2.71(2.57-2.85)	5.77(5.30-6.30)	1.47(1.34-1.61)	3.83(3.48-4.2)

### Cancer risk relative to the general population: all transplant recipients

After a total of 34,253 person-years of follow-up, 388 malignancies in recipients were identified during follow-up, corresponding to an overall cancer incidence of 1132.76 (95% CI, 1020.04-1245.47) cases per 100,000 person-years. Compared with the general population, the cancer SIR was 3.01 (95% CI, 2.73-3.33), and EAR was 756.4 (95% CI, 669.7-854.4) per 100,000 person-years. SIRs were significantly elevated for almost all sites including cancers of the oropharynx, gastrointestinal tract (liver and biliary tract), urinary tract, lung, bone/connective tissue/skin, and lymphoid/hematopoietic systems (*P* < .001), except for breast cancer. In particular, Kaposi sarcoma was associated with an SIR of 40.00 (95% CI, 10.00-159.94). The risk for Kaposi sarcoma; cancers of the urinary tract; lymphoid and hematopoietic malignancies; and cancer of bone, connective tissue, and skin was very high in transplant patients (Figure 2).

### Cancer risk relative to the general population: by transplanted organ

The incidence of cancer was highest in patients receiving liver transplantation (1361.4 cases per 100,000 person-years) followed by those receiving kidney (1101.2 per 100,000 person-years), heart (incidence, 878.4 cases per 100,000 person-years), and lung transplantation (728.9 cases per 100,000 person-years). The overall SIRs were 3.37 (95% CI, 2.81-4.05) for liver transplant recipients, 3.33 (95% CI, 2.93-3.79) for kidney transplant recipients, 1.82 (95% CI, 0.45-7.27) for lung transplant recipients, and 1.65 (95% CI,1.21-2.24) for heart transplant recipients.

We observed a pronounced risk of cancer at the respective sites of transplantation. Heart transplantation was associated with highest risk for lymphoid and hematopoietic malignancy (SIR, 4.55; 95% CI, 1.89-10.92), followed by lung cancer (SIR, 2.86; 95% CI, 1.54-5.31) (Table 2). In lung recipients, only two malignancies were diagnosed, one of which had a lymphoid and hematopoietic malignancy, and the other had cancer in the bone, connective tissue, and skin. Patients receiving kidney transplantation were associated with an SIR of 10.93 (95% CI, 9.20-12.99) for urinary tract malignancies, among which bladder cancer was the most common. Kidney recipients also had a higher risk for developing lymphoid and hematopoietic malignancies, and cancers of bone, connective tissue, skin; the liver and biliary tract, and other (Table 3). Liver recipients were eight-fold more likely to develop liver and biliary tract cancer; six-fold more likely to develop cancer of bone, connective tissue, and skin, and lymphoid and hematopoietic malignancy; three-fold more likely to develop cancer of lip, oral and pharynx, and urinary tract cancer; and two-fold more likely to develop gastrointestinal cancer (Table 4).

**Table 2:** Risk of malignancies in heart or lung transplant recipients

	Heart or Lung			Incidence/100,000^a^		
	Observed no.	Expected no	SIR (95% CI)	Observed	Expected	EAR/100000 Person-Years (95% CI)
All cancers	42	25.4	1.65(1.22-2.23)*	922.29	526.07	343.8(178.4 -509.2)
Oral cancer	2	2.3	0.87(0.22-3.48)	43.92	47.64	6.2(0.0 -88.9)
Salivary glands cancer	4	0.1	52.6(19.8- 140)*	87.84	2.07	80.8(21.5 -209.1)
Nasopharynx cancer	0	0	N/A	N/A	N/A	N/A
Esophagus cancer	1	0.8	1.31(0.18-9.30)	21.96	16.57	4.1(0.0 -84.9)
Gastric cancer	0	0	N/A	N/A	N/A	N/A
Colorectal cancer	7	3.5	1.99(0.95-4.17)	153.72	72.49	72.5(17.5 -197.0)
Liver cancer	1	4.7	0.21(0.03-1.49)	21.96	97.34	−76.6 (−203.1 to -19.5)
Gallbladder cancer	1	0.3	3.09(0.44-21.9)	21.96	6.21	14.5(0.1 -104.4)
Pancreatic cancer	0	0	N/A	N/A	N/A	N/A
Lung cancer	9	3.1	2.92(1.52-5.61)*	197.63	64.21	122.2(44.4 -267.6)
bone and articular cartilage cancer	0	0	N/A	N/A	N/A	N/A
connective and other soft tissue cancer	2	0.1	13.5(3.38-54.1)*	43.92	2.07	39.4(4.4 -146.3)
Melanoma	0	0	N/A	N/A	N/A	N/A
non-melanoma skin cancer	1	0.3	2.98(0.42-21.2)	21.96	6.21	14.5(0.1 -104.4)
Female breast cancer	1	1.2	0.83(0.12-5.89)	21.96	24.85	−4.1(−84.9 to 0.0)
Cervical cancer	2	1.5	1.33(0.33-5.33)	43.92	31.07	10.4(0.0 -96.8)
Urinary tract	0	0	N/A	N/A	N/A	N/A
Ovarian cancer	0	0	N/A	N/A	N/A	N/A
Prostate cancer	3	0.9	3.31(1.07-10.3)*	65.88	18.64	43.5(5.7 -152.9)
Bladder cancer	1	0.9	1.12(0.16-7.95)	21.96	18.64	2.1(0.0 -80.7)
Kidney cancer	0	0	N/A	N/A	N/A	N/A
Brain cancer	1	0.3	2.97(0.42-21.1)	21.96	6.21	14.5(0.1 -104.4)
Thyroid cancer	1	0.3	2.90(0.41-20.6)	21.96	6.21	14.5(0.1 -104.4)
Hodgkin's disease	6	0.7	8.55(3.84-19.0)*	131.76	14.50	109.8(37.1 -250.4)
Leukemia	0	0	N/A	N/A	N/A	N/A

^a^During 4828.27 person-years

**Table 3:** Risk of malignancies in kidney transplant recipients

	Kidney			Incidence/100,000^a^		
	Observed no.	Expected no	SIR (95% CI)	Observed	Expected	EAR/100000 Person-Years (95% CI)
All cancers	231	69.4	3.33(2.93-3.79)*	1101.18	330.83	770.3(651.6 -889.1)
Oral cancer	5	5.8	0.87(0.36-2.09)	23.84	27.65	−3.8(−24.9 to 0.0)
Salivary glands cancer	0	0	N/A	N/A	N/A	N/A
Nasopharynx cancer	2	2.8	0.71(0.18-2.84)	9.53	13.35	−3.8(−24.9 to 0.0)
Esophagus cancer	4	1.5	2.63(0.99-7.01)	19.07	7.15	11.9(2.0 -38.2)
Gastric cancer	5	3.3	1.52(0.63-3.65)	23.84	15.73	8.1(0.7 -32.1)
Colorectal cancer	4	7.9	0.51(0.19-1.36)	19.07	37.66	−18.6(−48.1 to -5.0)
Liver cancer	31	9.4	3.31(2.33-4.71)*	147.78	44.81	103.0(59.5 -146.4)
Gallbladder cancer	2	0.7	2.75(0.69-11.00)	9.53	3.34	6.2(0.3 -29.0)
Pancreatic cancer	3	0.9	3.28(1.06-10.2)*	14.30	4.29	10.0(1.3 -35.2)
Lung cancer	9	5.9	1.53(0.80-2.94)	42.90	28.13	14.8(3.2 -42.5)
bone and articular cartilage cancer	1	0.2	4.68(0.66-33.2)	4.77	0.95	3.8(0.0 -24.9)
connective and other soft tissue cancer	1	0.5	2.08(0.29-14.8)	4.77	2.38	2.4(0.0 -22.3)
Melanoma	1	0.2	5.07(0.71-36.0)	4.77	0.95	3.8(0.0 -24.9)
non-melanoma skin cancer	3	0.7	4.21(1.36-13.1)*	14.30	3.34	11.0(1.6 -36.7)
Female breast cancer	12	10.5	1.14(0.65-2.01)	57.20	50.05	7.2(0.5 -30.6)
Cervical cancer	3	10	0.30(0.10-0.93)*	14.30	47.67	−33.4(−68.8 to -13.4)
Urinary tract	2	1.2	1.66(0.42-6.64)	9.53	5.72	3.8(0.0 -24.9)
Ovarian cancer	0	0	N/A	N/A	N/A	N/A
Prostate cancer	4	1	4.03(1.51-10.70)*	19.07	4.77	14.3(2.9 -41.8)
Bladder cancer	63	1.6	38.60(30.10-49.40)*	300.32	7.63	292.7(219.5 -365.9)
Kidney cancer	9	0.3	28.80(15.00-55.30)*	42.90	1.43	41.5(18.7 -79.6)
Brain cancer	0	0	N/A	N/A	N/A	N/A
Thyroid cancer	11	2.1	5.32(2.95-9.61)*	52.44	10.01	42.4(19.3 -80.8)
Hodgkin's disease	11	1.8	6.16(3.41-11.1)*	52.44	8.58	43.9(20.3 -82.7)
Leukemia	2	1.1	1.83(0.46-7.32)	9.53	5.24	4.3(0.1 -25.7)

^a^During 20977.54 person-years

**Table 4:** Risk of malignancies in liver transplant recipients

	liver			Incidence/100,000^a^		
	Observed no.	Expected no	SIR (95% CI)	Observed	Expected	EAR/100000 Person-Years (95% CI)
All cancers	115	33.9	3.40(2.83-4.08)*	1361.44	401.33	960.1(751.2-1169.1)
Oral cancer	12	3.2	3.78(2.15-6.66)*	142.06	37.88	104.2(47.1-199.1)
Salivary glands cancer	0	0	N/A	N/A	N/A	N/A
Nasopharynx cancer	2	1.5	1.38(0.35-5.52)	23.68	17.76	5.9(0.0-55.3)
Esophagus cancer	3	1	3.14(1.01-9.74)*	35.52	11.84	23.7(2.9-85.5)
Gastric cancer	4	1.9	2.05(0.77-5.46)	47.35	22.49	24.9(3.2-87.4)
Colorectal cancer	7	4.4	1.59(0.76-3.34)	82.87	52.09	30.8(5.4-96.6)
Liver cancer	46	6	7.63(5.72-10.20)*	544.58	71.03	473.5(326.8-620.3)
Gallbladder cancer	1	0.4	2.33(0.33-16.50)	11.84	4.74	7.1(0.0-57.5)
Pancreatic cancer	0	0	N/A	N/A	N/A	N/A
Lung cancer	5	3.6	1.40(0.58-3.36)	59.19	42.62	16.6(1.0-74.0)
bone and articular cartilage cancer	1	0.1	9.31(1.31-66.10)*	11.84	1.18	10.7(0.2-63.9)
connective and other soft tissue cancer	1	0.2	5.19(0.73-36.90)	11.84	2.37	9.5(0.1-61.8)
Melanoma	0	0	N/A	N/A	N/A	N/A
non-melanoma skin cancer	3	0.4	7.45(2.40-23.10)*	35.52	4.74	30.8(5.4-96.6)
Female breast cancer	4	2.2	1.81(0.68-4.82)	47.35	26.04	21.3(2.2-81.7)
Cervical cancer	8	3.3	2.42(1.21-4.85)*	94.71	39.07	55.6(17.3-133.1)
Urinary tract	0	0	N/A	N/A	N/A	N/A
Ovarian cancer	1	0.3	3.45(0.49-24.50)	11.84	3.55	8.3(0.1-59.7)
Prostate cancer	4	0.7	5.94(2.23-15.80)*	47.35	8.29	39.1(8.9-109.1)
Bladder cancer	2	1	1.93(0.48-7.72)	23.68	11.84	11.8(0.3-66.0)
Kidney cancer	0	0	N/A	N/A	N/A	N/A
Brain cancer	0	0	N/A	N/A	N/A	N/A
Thyroid cancer	3	0.5	5.81(1.87-18.00)*	35.52	5.92	29.6(4.9-94.8)
Hodgkin's disease	7	0.9	7.83(3.73-16.40)*	82.87	10.65	72.2(26.8-156.2)
Leukemia	3	0.5	5.95(1.92-18.50)*	35.52	5.92	29.6(4.9-94.8)

^a^During 8446.92 person-years

### Analyses for cancers of the lymphoid/hematopoietic system, bone/connective tissue/skin system, liver/biliary tract and urinary tract

We conducted additional analyses for the four common sites of malignancies (Table 5). Among transplant recipients, except for cancers in bone, connective tissue, and skin, the risks of malignancies were higher in females than in males. No significant difference in SIR was observed in different age groups in the four cancer sites; however, the SIRs for lymphoma and liver, biliary, and urinary tract cancer were especially elevated among the youngest recipients.

**Table 5:** Risk of selected cancers in subgroups of transplant recipients

	Cancer Site			
	Lymphoid and hematopoietic	Bone, connective tissue and skin	Liver and biliary tract	Urinary tract
	**Observed Cases (Observed Incidence Rate/100 000 Person-Years)**			
Sex				
Male	14 (72.1)	9 (46.4)	63 (324.5)	53 (273.0)
Female	14 (94.5)	5 (33.8)	15 (101.3)	94 (634.5)
Age at transplant				
0-34	5 (44.5)	1 (8.9)	6 (53.4)	8 (71.2)
35-49	11 (80.5)	3 (22.0)	18 (131.7)	44 (322.1)
>=50	12 (128.4)	10 (107.0)	54 (577.7)	95 (1016.3)
Transplanted organ				
Kidney	13 (62.0)	6 (28.6)	31 (147.8)	129 (614.9)
Heart	5 (109.8)	2 (43.9)	1 (22.0)	6 (131.8)
Lung	1 (364.4)	1 (364.4)	-	-
Liver	9 (106.5)	5 (59.2)	46 (544.6)	15 (177.6)
	**Expected Cases (Expected Incidence Rate/100 000 Person-Years)**			
Sex				
Male	3.6 (18.5)	1.9 (9.8)	16.5 (85.0)	7.4 (38.1)
Female	2.0 (13.5)	1.1 (7.4)	3.5 (23.6)	12.7 (85.7)
Age at transplant				
0-34	0.5 (4.4)	0.2 (1.8)	0.3 (2.7)	0.5 (4.4)
35-49	1.3 (9.5)	0.7 (5.1)	3.6 (26.3)	4.7 (34.4)
>=50	3.8 (40.7)	2.2 (23.5)	16.1 (172.2)	14.9 (159.4)
Transplanted organ				
Kidney	2.9 (13.8)	1.6 (7.6)	9.4 (44.8)	11.8 (56.2)
Heart	1.1 (24.2)	0.6 (13.2)	4.5 (98.8)	3.3 (72.5)
Lung	0	0	0	0
Liver	1.5 (17.8)	0.8 (9.5)	6.0 (71.0)	4.8 (56.8)
	**Standardized Incidence Ratio (95% CI)**			
Sex				
Male	3.89(2.30-6.57)	4.74(2.46-9.10)	3.82(2.98-4.89)	7.16(5.47-9.37)
Female	7.00(4.15-11.82)	4.55(1.89-10.92)	4.29(2.58-7.11)	7.40(6.05-9.06)
Age at transplant				
0-34	10.00(4.16-24.03)	5.00(0.70-35.5)	20.00(8.99-44.52)	16.00(8.00-31.99)
35-49	8.46(4.69-15.28)	4.29(1.38-13.29)	5.00(3.15-7.94)	9.36(6.97-12.58)
>=50	3.16(1.79-5.56)	4.55(2.45-8.45)	3.35(2.57-4.38)	6.38(5.21-7.80)

## DISCUSSION

In this nationwide cohort population-based study, we found a three-fold risk of de novo cancer for heart, lung, kidney, and liver transplant recipients when compared with the general population. The increas in the absolute risk was significant, corresponding to 756.4 cases per 100,000 person-years. The risk was increased in a variety of site-specific cancers, with a distinct pattern specific to different kinds of organ transplantation. An elevated risk of lymphoid and hematopoietic malignancies was consistent in patients in all kinds of solid organ transplantation. Infection-related cancer, particularly Kaposi sarcoma, is associated with the highest SIR, despite a low EAR, because of the rarity of this malignancy. In addition, transplant recipients are also exposed to a three-fold greater risk of death. Our study confirms that patients receiving solid organ transplantation and subsequent immunosuppression are exposed to a higher risk of cancer, particularly infection-related and lymphoid and hematopoietic malignancies, and overall mortality.

The SIR of Kaposi sarcoma at 40 in transplant recipients was the highest among various de novo malignancies. In other population-based studies, the risk of Kaposi sarcoma in transplant recipients could be up to 200-fold [8, 9, 14]. Elevated risk of de novo malignancies in the lymphoid and hematopoietic systems was also found in all transplant recipients in our study. Although the specific type of lymphoid cancer was not obtained from the database, the estimated high risk was supported by the literature.

Post-transplant lymphoproliferative disorders (PTLD) are recognized as the second most frequent and aggressive form of malignancy occurring in transplant recipients, and the incidence of PTLD in transplant recipients is approximately eight-fold higher than in the general population [3, 8, 20]. The incidence can increase up to 20% in children after combined heart and lung transplantation [21]. Recent studies have revealed that non-Hodgkin lymphoma (NHL) is especially common in thoracic organ recipients, possibly as a result of higher doses of immunosuppressants required to prevent graft rejection [14, 22], and the risk of NHL-related mortality was significantly increased [23, 24]. Among viral infections, EBV is the main cause of PTLD. When EBV-seropositive organs have been transplanted to seronegative recipients, these patients are at higher risk of developing PTLD, especially children, who experience primary EBV infection after transplantation [25–27]. In addition to EBV, other viruses such as polyomavirus, cytomegalovirus, and HCV have also been associated with PTLD development in EBV-negative patients [5].

As illustrated by other studies (14,28-30), skin cancer was the most frequent malignancy following transplantation, and the risk of non-melanoma skin cancer was significantly elevated in both heart and lung recipients with an excess risk of mortality compared with the general population [24]. This elevated risk in bone, connective tissue, and skin was observed in kidney and liver recipients in the present study, but not in heart recipients, which could be attributed to the finite number of de novo malignancies found in our heart and lung recipients. Whether or not similar risks would be observed in the Asian population requires further studies.

In consistency with prior population-based studies [7–9, 23], the risk of lung cancer was elevated in heart recipients. The elevated risk could be related to the underlying lung condition in the native lung when only one lung was transplanted, repeated infections, or chronic smoking [31, 32]. The small number of lung transplant recipients, of which only two were diagnosed with cancer, was one of the limitations of our study.

Our study could add to existing evidence that showed an increased risk of cancer in the urinary tract in kidney and liver recipients [9, 11, 12], often in association with acquired polycystic kidney disease [23]. Another explanation is the high prevalence of the use of Chinese herbal medicine in Taiwan. It has been suggested that some constituents in herbal medicine, such as aristolochic acid and heavy metal, are associated with an increased risk of urinary tract cancers [33, 34].

An almost eight-fold increase in hepatobiliary cancer in liver recipients was observed in the current study, much higher than that reported by Koshiol*, et al* [35]. This finding may be associated with the high prevalence of HBV and HCV infection in Taiwan, especially in patients with liver cirrhosis, which is the main cause of liver failure and subsequent need for liver transplantation. The high prevalence of HBV infection (about 15%-20%) in Taiwan [36] may explain the magnitude of increased cancer risk in organ transplantation recipients. However, to determine whether such increased risk in transplant recipients was entirely due to HBV or HCV infection requires further data analysis among HBV and HCV-negative transplant recipients and HBV and HCV-positive individuals in the general population.

Several limitations deserve discussion. Firstly, the case definitions of solid organ transplantation and cancers were based on recordings in the catastrophic illness registry and ICD-9 procedural codes, and diagnoses are subject to potential misclassification. However, organ transplantation is a major procedure that requires specialist review for reimbursement from the NHI, which ensures the validity of our case definition. A catastrophic illness certificate for cancer generally requires information pathology and relevant clinical information for formal approval by the insurance administration.

On the other hand, the strengths of this population-based study included its large size and the representative sampling of all Taiwanese transplant recipients, allowing an overview and comparison of cancer risk across transplanted organs in the Asian population. Our choice to stratify patients based on gender, age, socioeconomic status, and transplanted organ provided major demographic and clinical characteristics related to cancer risk. However, the major limitations included the lack of relevant laboratory data such as specific cancer type and staging. In addition, cancer-related mortality may provide further information on cancer risk, but only the all-cause mortality rate was determined in the current study.

In conclusion, our study quantified the relative and absolute risk of de novo cancer among transplant recipients compared with the risk in the general population. We also identified a higher risk of specific cancers including hepatobiliary cancer for liver recipients, urinary tract cancer for kidney recipients, and lymphoid or hematopoietic cancer for cardiothoracic recipients. An adjustment in post-transplant immunosuppressants, a change in lifestyle (eg, smoking or alcohol cessation), and regular cancer screening in solid organ transplant recipients may altogether serve to decrease cancer risk.

## MATERIALS AND METHODS

This study was approved by the institutional review board of the Chang Gung Memorial Hospital, Taiwan (IRB 104-6697B) and the National Health Research Institute, the data holder for the NHI research database. All personal information was fully encrypted; therefore, patient informed consent was exempted.

### Study population

**Figure 1:**
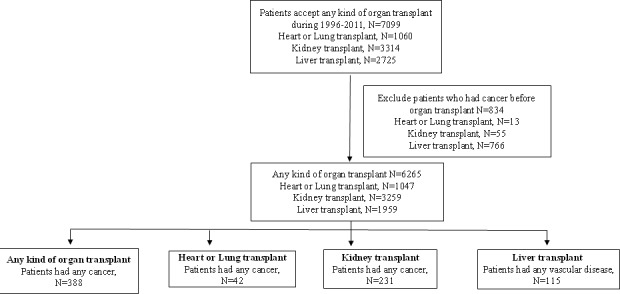
Flow chart of including organ transplant patients in inpatient file during 1996-2011

A cohort of all NHI patients registered in the Taiwan NHI database who received heart, lung, kidney, or liver transplantation between 1996 and 2011 was established. The NHIRD included all insurance claims data for the Taiwan NHI program. The NHI coverage rate was over 99.5% in 2010 [37]. The NHIRD was comprised of information about gender, date of birth, place of residence, insurance details (employment categories, sum of insurance amount, enrolment, and discharge date), dates of inpatient and outpatient visits, medical diagnoses, medical expenditures, prescription details, operations, and procedures. We excluded individuals without a valid insurance status, patients who did not undergo their organ transplantation in Taiwan, and patients who had cancer before receiving organ transplantation. The patients were followed until cancer developed, death occurred, or December 31, 2012, whichever came first (Figure 1).

**Figure 2:**
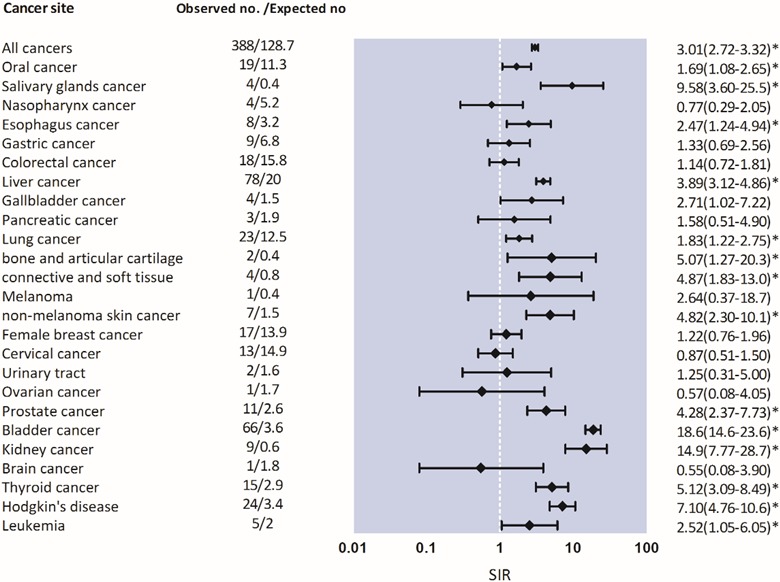
Risk of malignancies in heart, kidney, liver, lung transplant recipients

### Defining solid organ transplant and linkage with cancer

We used the International Classification of Diseases, Ninth Revision (ICD-9) codes for heart (37.5), lung (33.5), kidney (55.69), and liver transplantation (50.5); and the ICD-9 procedure codes for heart (V42.1), lung (V42.6), kidney (V42.0), and liver transplantation (V42.7) to identify patients who received these procedures in Taiwan between 1996 and 2011. The ICD-9 codes for cancer ranged between 140 and 208. In Taiwan, patients with cancer and/or received organ transplantation were entitled to a medical co-payment waiver. Diagnostic information of cancer and evidence of organ transplantation were sent to the insurance administration for a review by commissioned expert panels before waivers were approved. For a successful application of co-payment waivers, a pathology report supporting a specific type of cancer was a prerequisite for cancers, whereas operation records of solid organ transplantation were required for organ transplantations. The Registry for Catastrophic Illness Patients, a subset of NHIRD, contained information on these patients, including unique personal identification, diagnosis, demographics, application date, diagnosing physician, and hospital and other administrative data. We used this registry to identify patients with different types of cancer and those who received heart, lung, kidney or liver transplantation.

To ascertain the validity of cancer diagnoses in the NHI database, we linked the NHI with the National Cancer Registry, which served as the reference standard. We identified 835,967 incident cases of cancer between 2001 and 2012, as indicated by a catastrophic illness certificate for prostate cancer. Agreement between the NHI database and National Cancer Registry was good, with a sensitivity of 0.91, a specificity of 0·99, a positive predictive value of 0.94, and a negative predictive value of 1.

### Statistical analysis

Crude incidence rates of cancers were calculated as the total number of cancers during the follow-up time divided by person-years at risk. Person-years at risk was defined as the sum of patients from the date of organ transplantation to cancer occurrence, death, deregistration, or December 31, 2012, whichever came first. For age-specific rates, patient age was shifted along with calendar year because these ages contributed data to successive five-year age groups over the follow-up period.

To measure the relative risk of cancer in patients receiving transplantation when compared with the general population, we calculated an SIR for all types of cancers and specific cancer type. SIRs were computed as the ratios of observed numbers of cancers to the expected number of cancers on the basis of age-specific incidence rates in five-year age intervals of the general population, which were calculated by dividing the number of new cancer cases in a specific calendar year by the total person-years of the at-risk population. We performed additional analyses for the four common cancers for which SIRs were significantly elevated (lymphoid and hematopoietic cancers; cancers of bone, connective tissue and skin; cancers of the liver and biliary tracts; and cancers of the urinary tract).

We computed the absolute cancer risk in patients receiving organ transplantation compared with the general population by calculating excess absolute risk (EAR: observed incidence minus expected incidence). We also calculated the SMR for all-cause mortality. The SMR was the ratio of the observed number of deaths among organ transplantation recipients to the expected number of deaths in each gender and five-year age group by the corresponding national mortality rates in Taiwan. The mortality was defined by the permanent deregistration from the NHI. The 95% confidence intervals (CIs) for the SIR, EAR, and SMR were calculated, assuming a Poisson distribution. All tests of statistical hypothesis were based on the two-sided 5% level of significance. All analyses were performed using SAS v. 9.3 (SAS institute, Cary, NC).

## References

[1] Vajdic CM, van Leeuwen MT (2009). Cancer incidence and risk factors after solid organ transplantation. Int J Cancer.

[2] Cobucci RN, Saconato H, Lima PH, Rodrigues HM, Prudencio TL, Junior JE, Giraldo PC, Goncalves AK (2012). Comparative incidence of cancer in HIV-AIDS patients and transplant recipients. Cancer Epidemiology.

[3] Grulich AE, van Leeuwen MT, Falster MO, Vajdic CM (2007). Incidence of cancers in people with HIV/AIDS compared with immunosuppressed transplant recipients: a meta-analysis. Lancet.

[4] Grulich AE, Vajdic CM (2015). The epidemiology of cancers in human immunodeficiency virus infection and after organ transplantion. Semin Oncol.

[5] Piselli P, Verdirosi D, Cimaglia C, Busnach G, Fratino L, Ettorre GM, De Paoli P, Citterio F, Serraino D (2014). Epidemiology of de novo malignancies after solid organ transplantation: immunosuppression, infection and other risk factors. Best Pract Res Clin Obstet Gynaecol.

[6] Aberg F, Pukkala E, Hockerstedt K (2008). Risk of malignant neoplasms after liver transplantation: a population based study. Liver Transpl.

[7] Jiang Y, Villeneuve PJ, Wielgosz A, Schaubel DE, Fenton SS, Mao Y (2010). The incidence of cancer in a population based cohort of Canadian heart transplant recipients. Am J Transplant.

[8] Engels EA, Pfeiffer RM, Fraumeni JF, Kasiske BL, Israni AK, Snyder JJ, Wolfe RA, Goodrich NP, Bayakly AR, Clarke CA, Copeland G, Finch JL, Fleissner ML (2011). Spectrum of cancer risk among US solid organ transplant recipients. JAMA.

[9] Collett D, Mumford L, Banner NR, Neuberger J, Watson C (2010). Comparison of the incidence of malignancy in recipients of different types of organ: a UK Registry audit. Am J Transplant.

[10] Quinlan SC, Landgren O, Morton LM (2010). Hodgkin lymphoma among US solid organ transplant recipients. Transplantation.

[11] Villeneuve PJ (2007). Cancer incidence among Canadian kidney transplant recipients. Am J Transplant.

[12] Birkeland SA, Storm HH, Lamm LU, Barlow L, Blohme I, Forsberg B, Eklund B, Fjeldborg O, Friedberg M, Frodin L (1995). Cancer risk after renal transplantation in the Nordic countries, 1964-1986. Int J Cancer.

[13] Hall EC, Segev DL, Engels EA (2013). Racial/ethnic differences in cancer risk after kidney transplantation. Am J Transplant.

[14] Na R, Grulich AE, Meagher NS, McCaughan GW, Keogh AM, Vajdic CM (2013). Comparison of De Novo cancer incidence in Australian liver, heart and lung transplant recipients. Am J Transplant.

[15] Acuna SA, Fernades KA, Daly C, Hicks LK, Sutradhar R, Kim SJ, Baxter NN (2016). Cancer mortality among recipients of solid organ transplantation in Ontario, Canada. JAMA Oncol.

[16] Adami J, Gabel H, Lindelof B, Ekstrom K, Rydh B, Glimelius B, Ekbom A, Adami HO, Granath F (2003). Cancer risk following organ transplantation: a nationwide cohort study in Sweden. Br J Cancer.

[17] Serraino D, Piselli P, Angeletti C, Minetti E, Pozzetto A, Civati G, Bellelli S, Farchi F, Citterio F, Rezza G, Franceschi S, Busnach G (2005). Risk of Kaposi's sarcoma and of other cancers in Italian renal transplant patients. Br J Cancer.

[18] Miyazaki T, Oto T, Okumura M, Date H, Shiraishi T, Okada Y, Chida M, Kondo T, Nagayasu T (2016). De novo malignancy after lung transplantation in Japan. Gen Thorac Cardiovasc Surg.

[19] Tanaka S, Chen-Yoshikawa TF, Yamada T, Hijiya K, Motoyama H, Aoyama A, Date H (2016). Malignancies after living donor and cadaveric lung transplantations in Japanese patients. Surg Today.

[20] Dror Y, Greenberg M, Taylor G, Superina R, Hebert D, West L, Connolly B, Sena L, Allen U, Weitzman S (1999). Lymphoproliferative disorders after organ transplantation in children. Transplantation.

[21] Na R, Grulich AE, Meagher NS, McCaughan GW, Keogh AM, Vajdic CM (2013). De novo cancer-related death in Australia liver and cardiothoracic transplant recipients. Am J Transpl.

[22] Hall EC, Pfeiffer RM, Segev DL (2013). Cumulative incidence of cancer after solid organ transplantation. Cancer.

[23] Dharnidharka VR, Lamb KE, Gregg JA, Meier-Kriesche HU (2012). Associations between EBV serostatus and organ transplant type in PTLD risk: an analysis of the SRTR National Registry Data in the United States. Am J Transplant.

[24] Opelz G, Daniel V, Naujokat C, Dohler B (2009). Epidemiology of pretransplant EBV and CMV serostatus in relation to post transplant non-Hodgkin lymphoma. Transplantation.

[25] Minai OA, Shah S, Mazzone P, Budev MM, Sahoo D, Murthy S, Mason D, Pettersson G, Mehta AC (2008). Bronchogenic carcinoma after lung transplantation: characteristics and outcomes. J Thorac Oncol.

[26] Engels EA (2008). Inflammation in the development of lung cancer: epidemiological evidence. Expert Rev Anticancer Ther.

[27] Secnikova Z, Gopfertova D, Hoskova L, Hercogova J, Dzambova M, Jirakova A, Rajska L, Rob F, Smerhovsky Z (2015). Significantly higher incidence of skin cancer than other malignancies in patients after heart transplantation. A retrospective cohort study in the Czech Republic. Biomed Pap Med Fac Univ Palacky Olomouc Czech Repub.

[28] Krynitz B, Edgren G, Lindelof B, Baecklund E, Brattström C, Wilczek H, Smedby KE (2013). Risk of skin cancer and other malignancies in kidney, liver, heart and lung transplant recipients 1970-2008-a Swedish population based study. Int J Cancer.

[29] Awald FO, Brown M (2011). Skin cancer in solid organ transplant recipients: advances in therapyand management: part I. Epidemiology of skin cancer in solid organ transplant recipients. J Am Acad Dematol.

[30] Lebbe C, Legendre C, Frances C (2008). Kaposi's sarcoma in transplantation. Transplant Rev.

[31] Pierangeli A, Antonelli G, Gentile G (2015). Immunodeficiency-associated viral oncogenesis. Clin Microbiol Infect.

[32] Fernberg P, Edgren G, Adami J, Ingvar A, Bellocco R, Tufveson G, Hoglund P, Kinch A, Simard JF, Baecklund E, Lindelof B, Pawitan Y, Smedby KE (2011). Time trends in risk and risk determinants of non-Hodgkin lymphoma in solid organ transplant recipients. Am J Transplant.

[33] Hellstrom VC, Enstrom Y, von Zur-Muhlen B, Hagberg H, Laurell A, Nyberg F, Backman L, Opelz G, Dohler B, Holmberg L, Tufveson G, Enblad G, Lorant T (2016). Malignancies in transplanted patients: multidisciplinary evaluation and switch to mTOR inhibitors after kidney transplantation-experiences from a prospective, clinical, observational study. Acta Oncol.

[34] Cheung CY, Lam MF, Lee KC, Chan GS, Chan KW, Chau KF, Li CS, Chan TM, Lai KN (2011). Renal cell carcinoma of native kidney in Chinese renal transplant recipients: a report of 12 cases and a review of the literature. Int Urol Nephrol.

[35] Koshiol J, Pawlish K, Goodman MT, McGlynn KA, Engels EA (2014). Risk of hepatobiliary cancer after sold organ transplant in the United States. Clin Gastroenterol Hepatol.

[36] Department of Health and Welfare, Executive Yuan, Taiwan, ROC (2008). Public health report.

[37] Bureau of National Health Insurance, D.o.H., Executive Yuan, The National Health Insurance Statistics (2010). http://www.nhi.gov.tw/english/index.aspx?menu=8&menu_id=30&webdata_id=0&WD_ID=30.

